# Case of Gunshot Wound of Liver and Lung

**Published:** 1894-11

**Authors:** Thomas S. K. Morton

**Affiliations:** Professor of Surgery in the Philadelphia Polyclinic


### CASE OF GUNSHOT WOUND OF LIVER AND LUNG.

By THOMAS S. K. MORTON, M. D.

Professor of Surgery in the Philadelphia Polyclinic.

A boy, aged nine and a half years, was admitted to the Pennsylvania Hospital, September 11, 1894, with a history that he had been shot by a 32-calibre revolver at short range, but a few moments previously. I saw him almost at once after admission, and found a bullet wound one-and-a-half inches below and half an inch to the left of the ensiform cartilage. He was not especially shocked. Was said to have vomited considerable blood, and complained of great pain in the epigastrium. Abdomen not distended.

Ether was administered and perforation of the abdominal cavity proved by enlarging the bullet wound slightly and passing a probe. Having thus made certain that the peritoneum had been entered, an incision was made in the median line from the ensiform cartilage downward for four inches. Upon laying open the peritoneum much fluid, blood and some large clots flowed out. It was found that the ball had passed through the right lobe of the liver two-and-a-half inches behind the anterior margin, then emerged just above the gastro-hepatic omentum, had almost totally destroyed the lobus spigelii, then had torn a large hole in the lesser omentum, again perforated the peritoneum, struck the first lumbar vertebra and became lost. Blood welled up in large quantities from the posterior peritoneal opening, mostly venous, but partially arterial. A finger tip only could be passed into this wound. There was no wound of stomach or intestines. The wound of the right lobe, as well as that of the spigelian lobe of the liver, was not bleeding. A column of iodoform gauze was carried down so as to block the wounds of the lesser omentum and posterior layer of the peritoneum, and at the same time tc press upon the mutilated spigelian lobe and posterior or exit wound of the right lobe of the liver. The packing was continued and brought out through the parietal wound. The wound of entrance into the right lobe was not interfered with. The abdominal wound was not closed round the gauze drain after copious irrigation of the surroundings with hot salt solution. As it was suspected that much blood had gravitated into the pelvis and lower portions of the abdomen, which could not readily be washed out by irrigation from above, it was determined to make a small opening above the pubis for that purpose and to put in a drain-tube. Accordingly a half inch incision was made just above symphysis, and much fluid blood and clots were washed out through it by means of a long irrigator tube. A glass drain was carried through this wound down to the bottom of the pelvis, to serve as an index should further hemorrhage take place into the abdomen. But, despite all efforts to the contrary, the lad died in a few hours.

At the post-mortem examination it was discovered that the ball had passed between the aorta and vena cava, and perforated the right crus of the diaphragm before striking the first lumbar vertebra. In the latter it cut a large groove, and was deflected upward and outward throgh the pleura, and into the substance of the right lung, where it was found imbedded. The lower lobes of this lung were distended by blood, and over a quart in addition filled the pleural sac. No wounds of other viscera were discovered. There was no blood in the abdominal cavity.

Upon careful inquiry after death, I ascertained that the reported vomiting of blood had been incorrect; that in reality he had coughed up and not vomited it. No especial examination of the chest was made before operation, and no signs appeared to call attention to that locality. But the bleeding thereinto was unquestionably the immediate cause of death. Had the ball not wounded the lung, I believe that the boy would have had a very good chance of recovery afforded him by the operation.

DISCUSSION.

Dr. Edward Martin: Aside from other considerations the fact is worthy of note that Drs. Le Conte, Steinbach and Morton have been willing to report cases of cœliotomy for gunshot wound involving the abdominal contents, in which the result was not always favorable. It is certainly the exception to find any but the successful cases reported. Hence the results of a statistical study of this subject are most misleading. Thus in the admirable tabulation by Dr. T. S. K. Morton, published some years ago, the percentage of recovery after surgical intervention was so favorable that the inference as to the duty of every surgeon to operate at once on all these cases was direct. When all the cases operated on in a given section, New York, for instance, were collected, including those which were not published, it was found that the mortality was about the same for the cases treated expectantly, i. e., where operation was performed.

Reclus and Nougues, exploiting their sides of the subject, i.e., conservative treatment, deduced as the result of their statistical study the fact that the mortality after penetrating gunshot wounds of the abdomen is about twenty-five per cent. This is manifestly misleading, the result being due to the fact that only the rare successful cases of conservative treatment are published.

It still remains an open question as to whether a patient with a penetrating gunshot wound of the belly has not a better chance for recovery without operation, unless there should happen to be present a skilled abdominal surgeon, armed with all the appliances of his art.

The importance of this consideration of the question rests upon the fact that the country doctor or country surgeon whose experience has been limited, and who is unable to secure the timely help of a specialist, need not feel that in treating penetrating gunshot wounds of the belly conservatively, he is unfaithful to his trust.

As to the diagnosis of these cases, i. e., the diagnosis of penetration, unless this is absolutely assured by leakage of fæces or gas, for instance, or by bloody vomiting, or by purging of blood, or other pathognomonic symptoms, or by passing of the probe into the abdominal cavity, the fact of penetration should be definitely ascertained by exploratory incision in the line of the bullet wound before proceeding to a formal cœliotomy in the middle line.

The importance of bearing this in mind was well illustrated by a case which I saw in consultation with Dr. Edward Bidwell, of Vineland, N. J. The patient was a very fat woman, who had been shot from directly in front with a 32-calibre pistol. The wound of entrance was a quarter of an inch below the level of the umbilicus and two inches to the left. The patient was vomiting, had a rapid pulse, was tympanitic, complained of peritoneal tenderness upon the left side of the abdomen. The probe passed obliquely downward and backward through about three inches of fat and stopped at the muscles. Apparently the case was a fairly clear one of penetration and visceral wound. Exploratory incision along the track of the ball showed that it ranged downward and backward, penetrated the abdominal muscles one and a half inches above Poupart's ligament, grazed the peritoneum, and buried itself in the muscles of the pelvis. The wound was cleaned and closed and the woman was purged. In this case cœliotomy might have proved most unfortunate.

For flushing out the abdominal cavity, the normal saline so-ution is very much better than plain distilled water. The latter causes a primary blanching and a secondary acute hyperæmia, as shown by repeated experiments on dogs by Dr. Hobart Hare and myself. The normal saline solution is perfectly bland and unirritating.

As to the method of closing the belly wound, there is wide diversity of opinion. Perhaps there is a growing tendency at present to first stitch the peritoneum with a continued silk suture, then complete the closure by stitches taken through fascia, muscles, and skin. In any event, except for buried sutures, silk-worm-gut should be used.

Dr. G. G. Davis: In a gunshot wound of the intestine the ball oftentimes perforates both sides of the wall, and it makes so many openings that, as Dr. Le Conte has pointed out, the most careful examination is necessary in order that none may be overlooked. This takes so long that, in order to save time, in some cases, it would be better to resect as does Murphy with his button, in preference to other methods, such as stitching each separate wound and especially circular enterorrhaphy.

Dr. John B. Roberts: Dr. Martin has spoken of the possible bad results of gunshot wounds of the abdomen, and said that the mortality is not greater in such cases when left to nature, with good nursing, than when treated by modern surgical methods. Of course, it is difficult to decide this, but my own impression is that it is far better to open the abdomen in all penetrating wounds. If the operation be done by modern methods, the result is, I believe, much better than can be obtained by expectant treatment. Under the old methods, nearly all penetrating wounds of the abdomen caused death. I recall, however, one case of gunshot wound that recovered when such abdominal operations as we now perform were unknown. The patient was treated expectantly and did not die. His recovery, however, was regarded by the surgeons who saw him as very unusual. I, personally, am strongly in favor of section as soon as it is proved that the ball has entered the abdominal cavity. I believe in cutting down upon the bullet track to establish the fact that the missile has entered the abdomen, as was done in Dr. Steinbach's case, and then making an incision in the middle line.

